# circGRHPR inhibits aberrant epithelial-mesenchymal transformation progression of lung epithelial cells associated with idiopathic pulmonary fibrosis

**DOI:** 10.1007/s10565-024-09839-8

**Published:** 2024-01-25

**Authors:** Wensi Wu, Zhi Wang, Huiying Zhang, Xiaojun Zhang, Hui Tian

**Affiliations:** 1 Department of Thoracic Surgery, Qilu Hospital of Shandong University, No. 107, Wenhua West Road, Lixia District, Jinan, 250012 People’s Republic of China; 2 Department of Anesthesiology, Sun Yat-Sen Memorial Hospital, No. 107, Yanjiang West Road, Yuexiu District, Guangzhou, 510120 People’s Republic of China; 3 Department of Anesthesiology, Qilu Hospital of Shandong University, No. 107, Wenhua West Road, Lixia District, Jinan, 250012 People’s Republic of China

**Keywords:** Circular RNA, Lung epithelial cells, Epithelial-mesenchymal transition, Idiopathic pulmonary fibrosis, Ubiquitination

## Abstract

Air pollution has greatly increased the risk of idiopathic pulmonary fibrosis (IPF). Circular RNAs (circRNAs) have been found to play a significant role in the advancement of IPF, but there is limited evidence of correlation between circRNAs and lung epithelial cells (LECs) in IPF. This research aimed to explore the influence of circRNAs on the regulation of EMT progression in LECs, with the objective of elucidating its mechanism and establishing its association with IPF. Our results suggested that the downregulation of circGRHPR in peripheral blood of clinical cases was associated with the diagnosis of IPF. Meanwhile, we found that circGRHPR was downregulated in transforming growth factor-beta1 (TGF-β1)–induced A549 and Beas-2b cells. It is a valid model to study the abnormal EMT progression of IPF-associated LECs in vitro. The overexpression of circGRHPR inhibited the abnormal EMT progression of TGF-β1-induced LECs. Furthermore, as the sponge of miR-665, circGRHPR released the expression of E3 ubiquitin-protein ligase NEDD4-like (NEDD4L), thus promoting its downstream transforming growth factor beta receptor 2 (TGFBR2) ubiquitination. It is helpful to reduce the response of LECs to TGF-β1 signaling. In summary, circGRHPR/miR-665/NEDD4L axis inhibited the abnormal EMT progression of TGF-β1-induced LECs by promoting TGFBR2 ubiquitination, which provides new ideas and potential targets for the treatment of IPF.

**Graphical Abstract:**

**Graphical headlights**

1. Downregulation of circGRHPR in peripheral blood is associated with clinical diagnosis of IPF.

2. circGRHPR inhibits the abnormal EMT progression of TGF-β1-induced LECs in vitro.

3. circGRHPR/miR-665/NEDD4L axis inhibits the abnormal EMT progression of TGF-β1-induced LECs by promoting ubiquitination of TGFBR2 in vitro.

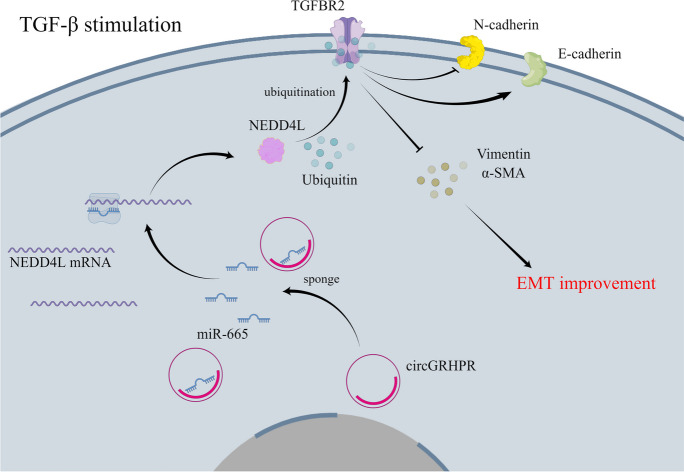

## Introduction

Idiopathic pulmonary fibrosis (IPF) is a long-term, advancing condition characterized by the development of fibrotic interstitial pneumonia. Air pollution, especially PM2.5, greatly increases the risk of IPF (Wang et al. 2022; Yang et al. 2020a, 2020b). The representative features of IPF include the proliferation of myofibroblasts and abundant deposition of extracellular matrix, which ultimately leads to irreversible lung tissue injury and respiratory failure (Li et al. 2020a). IPF is characterized by a tumor-like disease and exhibits an extremely unfavorable prognosis. After the diagnosis of IPF, the median survival period of the patient is only less than 3 years, with approximately 80% mortality rate within 5 years (Huang et al. 2022). Meanwhile, the incidence rate of IPF is increasing, and at least 3 million people around the world are facing serious risks of this disease (Cheng et al. 2022).

Timely diagnosis and intervention are very important for extending the lifespan for IPF individuals. However, the pathogenesis of IPF is complex and unclear at present. This leads to a lack of effective treatment options for IPF other than lung transplantation, and even nintedanib and pirfenidone only delay the progression of the disease (Yang et al. 2023). In addition to fibroblasts, IPF is also related to the pathological changes of lung epithelial cells (LECs), mainly including alveolar and bronchial epithelial cells (Fujishima et al. 2010; Cabrera et al. 2015). The latest researches have shown that alveolar epithelial cells produce myofibroblasts through abnormal epithelial-mesenchymal transition (EMT), which drives the fibrosis process of IPF (Song et al. 2011; Kim et al. 2009; Kim et al. 2021; Li et al. 2021). Bronchial epithelial cells contribute to airway remodeling in IPF through abnormal EMT progression (Wang et al. 2023). These processes are mainly co-regulated by transforming growth factor-beta1 (TGF-β1) signal (Pattarayan et al. 2018). In other words, finding effective ways to suppress the abnormal EMT of LECs will contribute to the treatment of IPF (Ptasinski et al. 2021).

Circular RNAs (circRNAs) are a unique category of long non-coding RNAs that arise through the process of reverse splicing from pre-messenger RNAs. circRNAs lack a poly(A) tail at the 3′ end and a cap structure at the 5′ end, forming a single-stranded closed-loop structure through end-to-end covalent bonding. The special structure of circRNAs makes them less susceptible to RNA exonuclease and more stable in expression, which is widely present in eukaryote organisms (Arnaiz et al. 2019; Xu et al. 2020; Zhang et al. 2023). In general, circRNAs function as miRNA sponges to alleviate the suppression of target genes and thereby regulate the development of diseases (Kristensen et al. 2019). Many studies have found that circRNAs inhibited the progression of various malignant tumors by regulating the EMT process (Wong et al. 2022; Pan et al. 2020; Shang et al. 2019). Meanwhile, there is some evidence that circRNAs can participate in the development of IPF as a novel biomarker (Li et al. 2020a; Li et al. 2018; Cheng et al. 2022). However, it is still unknown whether circRNAs are involved in the abnormal EMT progression of LECs in IPF. Overall, the function and mechanism of circRNAs in IPF remain largely unexplored.

We collected the peripheral blood samples to analyze the expression level of circRNAs in patients with IPF (Li et al. 2018) and further observed a downregulation of circular RNA hsa_circ_0001861 in TGF-β1-induced LECs in vitro. Hsa_circ_0001861 was derived from the exons 2–4 of glyoxylate reductase/hydroxypyruvate reductase (GRHPR), also named as circGRHPR in this study. Notably, circGRHPR is specific to humans and lacks homologous genes in mice or rats; therefore, our investigation primarily focused on its function and mechanism in vitro. Functionally, we discovered that circGRHPR suppressed the abnormal EMT progression of TGF-β1-induced LECs in vitro. Mechanistically, we found that circGRHPR acted as a sponge for miR-665 to released E3 ubiquitin-protein ligase NEDD4-like (NEDD4L), subsequently enhancing downstream ubiquitination of transforming growth factor beta receptor 2 (TGFBR2) and downregulating its expression. As TGFBR2 serves as a key mediator of TGF-β1 signaling and inhibiting its function or downregulating its expression has been shown to ameliorate IPF (Romero-Gallo et al. 2005; Marchal-Duval et al. 2023; Fierro-Fernandez et al. 2015), our study provides fundamental evidence elucidating the role and mechanism of circGRHPR in LECs. Additionally, we observed significantly decreased levels of circGRHPR in peripheral blood samples from IPF patients compared to healthy controls, suggesting its potential diagnostic relevance for IPF. Currently unexplored is the association between circGRHPR and the pathogenesis of IPF. Thus, our findings offer novel insights warranting further investigation into the involvement of circRNAs in IPF.

## Methods

### Participants and clinical samples

The scope of our study comprised 20 patients with IPF, who were admitted to Qilu Hospital at Shandong University (Jinan, China) between October 2022 and June 2023. Their diagnosis was confirmed using a comprehensive approach involving clinical evaluation, radiological imaging, and pathological examination according to the consensus criteria established by the American Thoracic Society/European Respiratory Society (Raghu et al. 2011). During the same period, we randomly selected 20 age-matched healthy people from the recruited volunteers as controls, excluding respiratory disease and smokers. Fresh peripheral blood samples were collected from all volunteers using a one-time vacuum sampling vessel containing EDTA (cat# 367,525, Corning, USA), and plasma was harvested after centrifugation. Total RNA was extracted from plasma samples by using TRIzol reagent (cat# 15,596,026, ThermoFisher, USA) following the guidelines provided by the manufacturer.

### Bioinformatic analysis

The gene expression omnibus database was utilized to obtain microarray data related to IPF. The GSE102660 dataset contained circRNA expression data of 3 pairs of IPF cases and controls. The GSE21411 dataset contained miRNA expression data from 9 IPF cases/6 controls. The GSE53845 and GSE101286 datasets contained mRNA expression data from 40 IPF cases/8 controls and 7 IPF cases/3 controls, respectively. The threshold used to identify genes with differential expression was adjust *P*-value < 0.05 and |log_2_fold-change|≥ 1. The Circinteractome (https://circinteractome.nia.nih.gov/index.html) (Dudekula et al. 2016), RNAalifold (http://rna.tbi.univie.ac.at/cgi-bin/RNAWebSuite/RNAalifold.cgi), and circMIR (https://www.bio-inf.cn/circmir/) databases were used to predict the relationship between circRNAs and miRNAs. The multiMiR package of R (version 4.2.3) software and targetscan (https://www.targetscan.org/vert_80/) database were used to predict the relationship between miRNAs and mRNAs. The results were visualized using R software.

### Cell culture

The cell line A549 (cat# CCL-185) represents human alveolar epithelial cells and the cell line Beas-2b (cat# CRL-3588) represents human bronchial epithelial cells, respectively. The A549 and Beas-2b cells were acquired from the American Type Culture Collection (USA) and cultured in Ham’s F-12K (Kaighn’s) Medium (cat# L450KJ, BasalMedia, CHN) and RMPI-1640 medium (cat# L210KJ, BasalMedia, CHN), respectively. All media were enhanced with a 10% concentration of fetal bovine serum (cat# A6901FBS, Invigentech, USA). Similar to previous studies (Li et al. 2020a; Wojcik-Pszczola et al. 2022), cells were starved overnight and further cultured in serum-depleted medium (1%) supplemented with TGF-β1 (10 ng/mL, cat# 10,804-HNAC, Sino Biological, CHN) for 48 h to induce the phenotype of EMT progression. All cells were incubated in a temperature-controlled environment at 37 °C with a CO_2_ concentration of 5%.

### cDNA synthesis and quantitative real-time PCR (qRT-PCR)

As described in our previous study (Wu et al. 2021a; Wu et al. 2021b), total RNA from A549 and Beas-2b cells in 6-well plates was extracted using FastPure Cell/Tissue Total RNA Isolation Kit V2 (cat# RC112-01, Vazyme, CHN). The total RNA samples were quantified using an ultramicroscopic spectrophotometer (cat# DS11 + , DeNovix, USA) to determine their concentrations. For circRNAs and mRNAs, cDNA synthesis was performed using the Hifair® III 1st Strand cDNA Synthesis Kit (cat# 11139ES60, Yeasen, CHN). For miRNAs, the All-in-One™ miRNA First-Strand cDNA Synthesis Kit 2.0 was utilized for the performance of cDNA synthesis (cat# QP113, GeneCopoeia, USA). LightCycler 480 II Real-Time PCR System (Roche) was used for quantitative quantification analysis. The internal control for circRNA and mRNA was 18S ribosomal RNA (18S rRNA), while U6 served as the internal control for miRNA expression. The 2^−ΔΔCt^ method was utilized to determine the relative expression levels. The sequences of primers are presented in Table 1.

**Table 1:** The sequence of primers

Genes	Sequence (5′-3′)
hsa_circ_0044226	F: CGAAATGCTATCAGAGTCAATCC
	R: CCTGAGGTGTTGTACATGCA
hsa_circ_0008898	F: GCTGGAGAGACTGCCTGTAA
	R: TCCGCGACTAAGTACAGCAA
hsa_circ_0004099	F: GCTCTACTCACAGCCATTATGC
	R: TCGTACTTGAAATGAAAGGGCT
hsa_circ_0035796	F: AAGGGAGAGAGTGGCCGATT
	R: AGTTCGAGTAAATGAATGCTCCT
hsa_circ_0044234	F: GGTGATTCAGCTTGCTTTACTCT
	R: CAAGGCTTCTTCTGAGTGTACAA
hsa_circ_0043278	F: GCTCTACTCACAGCCATTATGC
	R: TCGTACTTGAAATGAAAGGGCT
hsa_circ_0000977	F: GTCTCTACCCCAACTCAGGC
	R: TTCTCCGCAGCATCAGTTTG
hsa_circ_0088220	F: TCGTGAAGCAGAAGATGACG
	R: CTTGATTGGGCGTGAAGGAG
hsa_circ_0026933	F: AATACAGGAGGCTGCAAGGT
	R: ATGTTTCATTTCCCCACGCC
hsa_circ_0001861 (circGRHPR)	F: GTCCTGACAGATACCACCGC
	R: GCAGCATCCAGGATCCTCTT
18S rRNA	F: GGAGCCTGCGGCTTAATTTG
	R: CCACCCACGGAATCGAGAAA
GRHPR	F: ATGTCCTGACAGATACCACCG
	R: CGTGAGTCCATAGCCACACA
U6	F: CTCGCTTCGGCAGCACA
	R: AACGCTTCACGAATTTGCGT
miR-665	F: ACCAGGAGGCTGAGGCCCCT
miR-526b-5p	F: CTCTTGAGGGAAGCACTTTCTGT
NEDD4L	F: GACATGGAGCATGGATGGGAA
	R: GTTCGGCCTAAATTGTCCACT
GREM1	F: CGGAGCGCAAATACCTGAAG
	R: GGTTGATGATGGTGCGACTGT
SYT15	F: GTTCTCGGTGGAATATGAGCAG
	R: GCGTTTGGTCTTGGATTGGAG
AK4	F: TCACACGCCTAATGATGTCCG
	R: CGGCTGAGACGATCTTTAAGTG

### Gel electrophoresis and Sanger sequencing

The agarose (cat# 1110GR100, BioFroxx, GER), TAE buffer (cat# ST716, Beyotime, CHN), and gel red (cat# EZGR001, WSHTBio, CHN) were mixed, heated, and cooled to prepare a 1.5% agarose gel. The PCR products derived from complementary DNA (cDNA) and genomic DNA (gDNA) were subjected to electrophoresis at a voltage of 100 V and a duration of 40 min, followed by detection using an ultraviolet chemiluminescence instrument (cat# 1600, Tanon, CHN). GoldBand 100-bp DNA ladder (cat# 10507ES60, Yeasen, CHN) was used as a standard DNA marker for DNA size. TIANgel Midi Purification Kit (cat# DP219-03, Tiangen, CHN) was used to recover and purify PCR products separated in agarose gel, and further Sanger sequencing was performed by Genewiz Biotechnology Co. LTD (Tianjin, CHN).

### Actinomycin D experiment

A549 and Beas-2b cells were exposed to actinomycin D at a concentration of 2 μg/ mL in a 6-well plate (cat# GC16866, GlpBio, USA) for 0/12/16/20/22/24 h, respectively. The expression levels of circGRHPR and linear GRHPR were analyzed by qRT-PCR.

### RNase R treatment

Total RNA was isolated from harvested A549 and Beas-2b cells. Each 2 μg of total RNA underwent treatment with RNase R (3U/μg, cat# GE10003, GlpBio, USA) or enzyme-free water for a duration of 30 min at a temperature of 37 °C. The expression levels of circGRHPR and linear GRHPR were detected by qRT-PCR.

### Cell transfection

The overexpressed plasmid of circGRHPR (OE-circGRHPR) based on pGCMV vector and its negative control were purchased from GenePharma Pharmaceutical Technology Co. LTD (Shanghai, China). The overexpressed plasmid of NEDD4L (OE-NEDD4L) based on pCMV vector and its negative control were purchased from MiaoLing Plasmid Platform (Wuhan, China). The short interfering RNAs (siRNA) (si-circGRHPR, si-NEDD4L), miR-665 mimic or inhibitor, and negative controls (si-NC, NC-inhibitor) were produced by GenePharma Pharmaceutical Technology Co. LTD (Shanghai, China), and the sequences of oligonucleotides are shown in Table 2. When cells in the 6-well plate were cultured to 70% confluence, jetPRIME (cat# 101,000,046, Polyplus, FRA) reagent was used for transfection. Each well contained a total of 2 μg plasmid or oligonucleotides with a final concentration of 50 nM.

**Table 2:** The sequence of siRNA, probes, miRNA mimic, and inhibitor

Targets	Sequence (5′-3′)
si-circGRHPR#1	GAGGAAGUGAAGAACUGUGTT
si-circGRHPR #2	GGAAGUGAAGAACUGUGAGTT
si-NEDD4L#1	CGCCUUGACUUACCUCCAUTT
si-NEDD4L#2	GCGGAUGAGAAUAGAGAACTT
si-NC	UUCUCCGAACGUGUCACGUTT
miRNA inhibitor NC	CAGUACUUUUGUGUAGUACAA
miR-665 mimics	F: ACCAGGAGGCUGAGGCCCCU
	R: GGGCCUCAGCCUCCUGGUUU
miR-665 inhibitor	AGGGGCCUCAGCCUCCUGGU
Cy3-circGRHPR	CCACCTCACAGTTCTTCACTTCCTC
FAM-miR-665	AGGGGCCTCAGCCTCCTGGT
Biotin-circGRHPR	ACCTCACAGTTCTTCACTTCCTCGATGG
Biotin-miR-665	ACCAGGAGGCTGAGGCCCCT

### Fluorescence in situ hybridization

A549 and Beas-2b cells in a 24-well plate were fixed using 4% paraformaldehyde (cat# BL539A, Biosharp, CHN); then, the cells were pre-hybridized using a fluorescent in situ hybridization kit (GenePharma, CHN). Cy3-labeled circGRHPR probe (GenePharma, CHN) and FAM-labeled miR-665 probe (GenePharma, CHN) were hybridized with A549 and Beas-2b cells overnight at 37 °C. Then, after staining the 4′,6-diamidino-2-phenylindole (DAPI, cat# G1012, Servicebio, CHN) for a duration of 10 min, a positive fluorescence microscope (cat# Ni-U, Nikon, JPN) was used to observe and obtain images. The sequences of probes are presented in Table 2.

### Biotin-labeled probe RNA pull-down assay

The circGRHPR probe labeled with biotin and the negative control oligonucleotide probe were synthesized by GenePharma Pharmaceutical Technology Co. LTD (Shanghai, China). Subsequently, they were incubated with streptavidin magnetic beads (cat# HY-K0208, MedChemExpress, USA) for a duration of 2 h at room temperature. About 1 × 10^7^ A549 and Beas-2b cells were harvested in a 10-cm culture dish and fixed with 1% formaldehyde. Following that, the lysates were incubated with magnetic beads coated with the probe for one night in a 4 ℃ environment. After the beads were eluted, the obtained RNA samples were further reversed and the expression levels of miRNAs were detected by qRT-PCR. The sequences of biotin-labeled probes are presented in Table 2.

### Biotin-labeled probe RNA capture assay

The miR-665 probe labeled with biotin and the negative control oligonucleotide probe were synthesized by GenePharma Pharmaceutical Technology Co. LTD (Shanghai, China) and transfected into A549 and Beas-2b cells in culture dishes, respectively. Cells were harvested 48 h later, and the cell lysates were subjected to an overnight incubation with streptavidin magnetic beads (cat# HY-K0208, MedChemExpress, USA) in 4 °C condition. The expression level of circGRHPR was evaluated by qRT-PCR. The sequences of biotin-labeled probes are presented in Table 2.

### Dual-luciferase reporter assay

Dual-luciferase vector GP-miRGLO (GenePharma, CHN) was utilized to clone the wild-type and mutant binding sites of miR-665 in circGRHPR and the 3′ UTR of NEDD4L. The miR-665 mimics or negative control (GenPharma, CHN) was transfected into A549 and Beas-2b cells along with the dual-luciferase reporter plasmids. After 48 h, the luciferase activity of Renilla and Firefly was detected by Assay Kit (cat# 11402ES60, Yeasen, CHN).

### Western blot

As described in our previous study (Wu et al. 2021a), the lysates of A549 and Bsab-2b cells were prepared with the BCA protein quantification kit (cat# P0010, Beyotime, CHN) for protein samples. Relevant protein samples were subjected to SDS-PAGE electrophoresis, followed by subsequent transfer onto polyvinylidene fluoride membranes. The membranes were incubated with primary antibodies at 4 ℃ overnight. Primary antibodies include E-cadherin (1:500, cat# ET1607-75, Huabio, CHN), N-cadherin (1:1000, cat# ET1607-37, Huabio, CHN), Vimentin (1:5000, cat# ET1610-39, Huabio, CHN), α-smooth muscle actin (α-SMA, 1:1000, cat# ET1607-53, Huabio, CHN), NEDD4L (1:1000, cat# ET1611-42, Huabio, CHN), TGFBR2 (1:2000, cat# ER1917-66, Huabio, CHN), and glyceraldehyde-3-phosphate dehydrogenase (GAPDH, 1:10,000, cat# AB0037, Abway, CHN). After incubating with HRP-conjugated antibody (1:50,000, cat# ET1610-39, Huabio, CHN) for 60 min in normal temperature environment, the signal of protein band membrane was scanned by chemiluminescence instrument (cat# 5200, Tanon, CHN).

### Coimmunoprecipitation (Co-IP)

A549 and Beas-2b cells were lysed using Cell Lysis Buffers for Western and IP (cat# P0013J, Beyotime, CHN). The supernatants were incubated with TGFBR2 antibody (1:100, cat# 66,636–1-Ig, Proteintech, CHN) or IgG antibody (1:100, cat# AC011, Abclonal, CHN) for a duration of 6 h at room temperature. The mixtures were further mixed with Protein A/G Magnetic Beads (cat# HY-K0202, MedChemExpress, USA) and subjected to overnight incubation at 4 ℃. After that, the beads were washed and the antigen–antibody complex was eluted for western blotting to evaluate the expression of ubiquitin (1:1000, cat# ET1609-21, Huabio, CHN).

### Statistical analysis

All data were independently presented as the mean ± standard deviation (SD) from a minimum of three repetitions. The data was subjected to statistical analysis using software packages such as GraphPad Prism (version 9.0, USA) and R (version 4.2.3) software. The Student *t* test and one-way analysis of variance with Bonferroni correction were used for the analysis. The diagnostic value of circGRHPR was assessed using the receiver operating characteristic (ROC) curve. A statistically significant difference was determined when the *P*-value < 0.05.

## Results

### Identification and characterization of circGRHPR in abnormal EMT progression of IPF-associated LECs

**Fig. 1:**
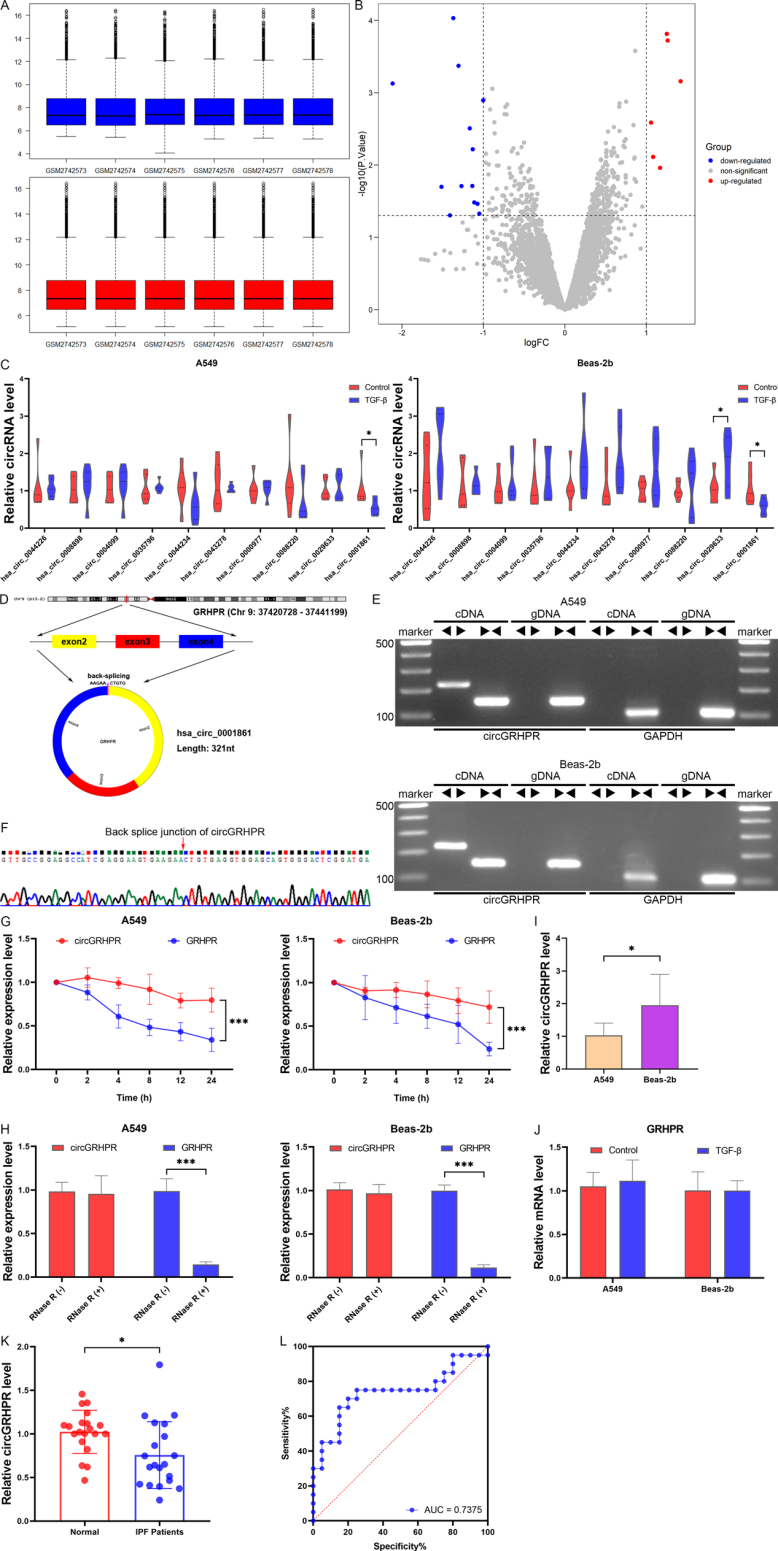
Identification and characteristics of circGRHPR in LECs and clinical patients. **A** Data standardization of microarray data GSE102660. **B**The volcano map showed differentially expressed circRNAs in GSE102660. **C** The expression of candidate circRNAs in TGF-β1-induced LECs. **D** The origin of hsa_circ_0001861. **E** Gel electrophoresis results of different PCR products. **F** The back splice junction of circGRHPR in Sanger sequencing. **G** The abundance of circGRHPR and GRHPR in actinomycin D–treated LECs. **H** The abundance of circGRHPR and GRHPR in RNase R–treated LECs. **I** The circGRHPR expression in two LEC cell lines. **J** The GRHPR expression in TGF-β1-induced LECs. **K** The circGRHPR expression in peripheral blood of healthy controls and IPF patients. **L** ROC curve analysis of circGRHPR for clinical diagnostic evaluation of IPF. Data represent as mean ± SD. *P < 0.05, ***P < 0.001

In order to identify the key circRNAs involved in the abnormal EMT procession of LECs associated with IPF, we standardized and analyzed the microarray data (GSE102660) from peripheral blood of IPF patients (Fig. 1A, B). The top five circRNAs with significantly upregulated expression were hsa_circ_0044226, hsa_circ_0008898, hsa_circ_0004099, hsa_circ_0035796, and hsa_circ_0044234. The top five circRNAs with significantly downregulated expression were hsa_circ_0043278, hsa_circ_0000977, hsa_circ_0088220, hsa_circ_0029633, and hsa_circ_0001861. We observed a significant decrease in the expression of hsa_circ_0001861 in both TGF-β1-induced models of LECs, which are associated with the progression of EMT (Fig. 1C). We searched the circBase database and found that hsa_circ_0001861 was derived from exons 2–4 of its parent gene GRHPR, with a length of 321 nucleotides, so we named it circGRHPR (Fig. 1D). The results of electrophoresis of PCR products showed that linear GRHPR in cDNA and gDNA could be amplified by convergent primers, while circGRHPR could only be amplified by the divergent primers in cDNA (Fig. 1E). The Sanger sequencing method was used to identify the PCR products sequence, and the result confirmed that the sequence of the reverse splicing site was the same as the annotation in circBase database (Fig. 1F). We treated A549 and Beas-2b cells continuously for 24 h with actinomycin D to inhibit transcription and found that circGRHPR had a greater half-life than the linear GRHPR mRNA (Fig. 1G). Meanwhile, we also found that circGRHPR had better resistance to RNase R than linear GRHPR mRNA (Fig. 1H). The cancerous epithelial cell line A549 exhibited a decreased expression of circGRHPR compared to the normal epithelial cell line Beas-2b (Fig. 1I). In addition, we observed no significant variation in the expression of GRHPR between the two types of LECs in TGF-β1-induced EMT progression models (Fig. 1J). These results demonstrated that circGRHPR is downregulated in the abnormal EMT progression of LECs and exhibits a loop structure.

We found that pulmonary function deteriorated significantly in patients with IPF compared to healthy controls. However, there were no notable disparities in age and gender among the two groups. The clinicopathological features of the patients are summarized in Table 3. To clarify the correlation between circGRHPR and clinical diagnosis of IPF, we found that the expression of circGRHPR in peripheral blood of IPF patients was significantly decreased compared with healthy controls (20 IPF cases and 20 healthy controls) (Fig. 1K). ROC curve results showed that the AUC of circGRHPR for IPF was 0.7375 (95% CI 0.5742–0.9008; *P* < 0.05). The sensitivity and specificity of circGRHPR for IPF diagnosis screening were 75.0% and 75.0%, respectively (Fig. 1L). All of the above results indicated that circGRHPR was associated with the EMT progression of IPF-related LECs.

**Table 3:** Clinicopathological characteristics of IPF patients and healthy controls

Characteristics	Controls (*n* = 20)	IPF patients (*n* = 20)	*P*-value
Age (years)	59.5 ± 15.26	67.55 ± 14.71	0.0976
Gender (male/female)	11/9	13/7	0.5309
FVC (% of predicted)	82.78 ± 4.355	61.16 ± 9.68	< 0.001
FEV1/FVC (% of predicted)	84.47 ± 3.936	82.15 ± 5.535	0.1342
TLC (% of predicted)	85.27 ± 5.935	71.2 ± 8.353	< 0.001
DLCO (% of predicted)	84.43 ± 4.606	62.21 ± 12.59	< 0.001
PaO_2_ (mmHg)	89.74 ± 3.04	64.69 ± 7.511	< 0.001
PaCO_2_ (mmHg)	37.46 ± 3.037	38.06 ± 3.505	0.5693

Values are expressed as the mean ± SD. *FVC* forced vital capacity, *FEV1/FVC* ratio of forced expiratory volume in the first second to forced vital capacity, *TLC* total lung capacity, *DLCO* diffusing capacity for carbon monoxide, *IPF* idiopathic pulmonary fibrosis

### circGRHPR inhibits abnormal EMT procession of TGF-β1-induced LECs in vitro

**Fig. 2:**
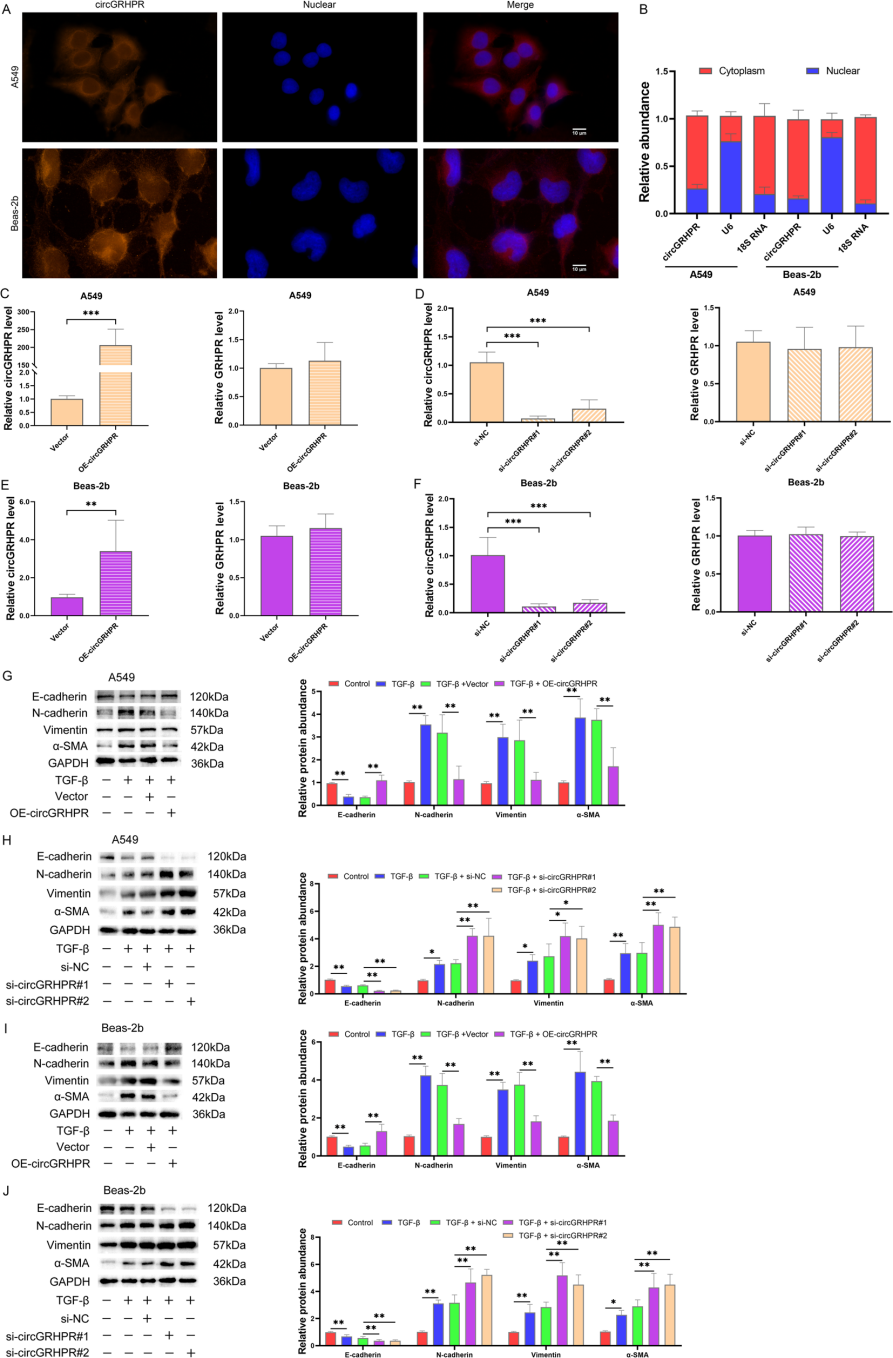
circGRHPR inhibits abnormal EMT procession of TGF-β1-induced LECs in vitro. **A** Subcellular localization of circGRHPR in LECs. **B** The abundance of circGRHPR in the nucleus and cytoplasm of LECs. **C** The abundance of circGRHPR and GRHPR after transfection with circGRHPR overexpressing plasmid in A549. **D** The abundance of circGRHPR and GRHPR after transfection with circGRHPR siRNA in A549. **E** The abundance of circGRHPR and GRHPR after transfection with circGRHPR overexpressing plasmid in Beas-2b. **F** The abundance of circGRHPR and GRHPR after transfection with circGRHPR siRNA in Beas-2b. **G** The effect of circGRHPR upregulation on EMT progression in A549. **H** The effect of circGRHPR downregulation on EMT progression in A549. **I** The effect of circGRHPR upregulation on EMT progression in Beas-2b. **J** The effect of circGRHPR downregulation on EMT progression in Beas-2b. All data were expressed as mean ± SD. *P < 0.05, **P < 0.01, ***P < 0.001

The cytoplasm of LECs was identified as the primary subcellular localization site for circGRHPR, according to the outcomes of FISH and subcellular localization assay (Fig. 2A, B). To explore the function of circGRHPR, we upregulated or downregulated circGRHPR in A549 and Beas-2b cells through overexpressed plasmid or siRNA, respectively. After transfection, the expression of circGRHPR in A549 and Beas-2b cells was successfully upregulated or downregulated without affecting the expression of linear GRHPR mRNA (Fig. 2C–F). The overexpression of circGRHPR inhibited the abnormal EMT progression of TGF-β1-induced LECs in vitro, while downregulating its expression significantly promoted the progression of EMT (Fig. 2G–J).

### circGRHPR binds to miR-665 in LECs

**Fig. 3:**
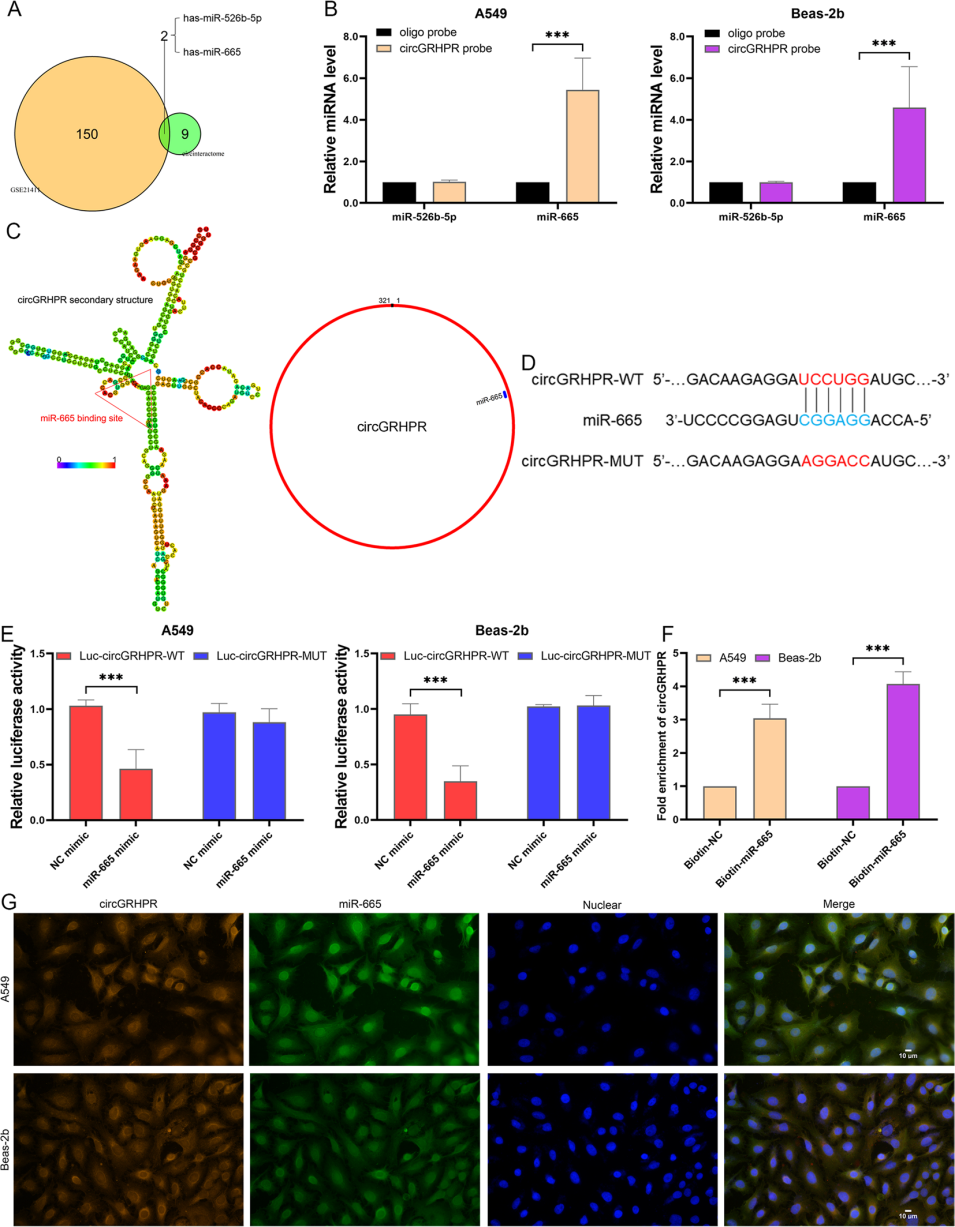
circGRHPR functions as a sponge for miR-665. **A** Prediction results of potential target miRNAs for circGRHPR. **B** RNA pull-down assay results of circGRHPR and miR-665 in LECs. **C**, **D** Prediction results of the secondary structure of circGRHPR and the binding site of miR-665. **E** Dual-luciferase reporter assay results of circGRHPR and miR-665 in LECs. **F** The abundance of circGRHPR captured by biotin-labeled miR-665 probes in LECs. **G** Subcellular localization of circGRHPR and miR-665 in LECs. Data represent as mean ± SD. ***P < 0.001

Normally, circRNAs localized in the cytoplasm can interact with miRNAs as RNA sponges (Hansen et al. 2013). To identify the miRNAs that interact with circGRHPR, we used the Circinteractome database for prediction and further screened the results using microarray data GSE21411. The results showed that miR-526b-5p and miR-665 were potential binding partners for circGRHPR (Fig. 3A). The RNA pull-down assay results showed that the circGRHPR probe labeled with biotin significantly enriched miR-665 in LECs compared to the oligonucleotide probe (Fig. 3B). We used RNAalifold and circMIR databases to analyze the targeted binding sequences of circGRHPR and miR-665 (Fig. 3C). On this basis, we further constructed luciferase reporter genes containing wild-type (WT) or mutant (MUT) circGRHPR sequences (Fig. 3D). Compared with NC mimic, miR-665 mimic significantly reduced the luciferase activity of circMBOAT2-WT reporter, but not the circGRHPR-MUT reporter (Fig. 3E). Furthermore, we found that circGRHPR can significantly enrich biotin-labeled miR-665, which further confirmed the interaction between circGRHPR and miR-665 (Fig. 3F). The results of FISH demonstrated that circGRHPR and miR-665 were co-localized in the cytoplasm of LECs (Fig. 3G). These results demonstrated that circGRHPR sponges miR-665 in LECs.

### circGRHPR/miR-665 targets NEDD4L to regulate EMT procession of TGF-β1-induced LECs in vitro

**Fig. 4:**
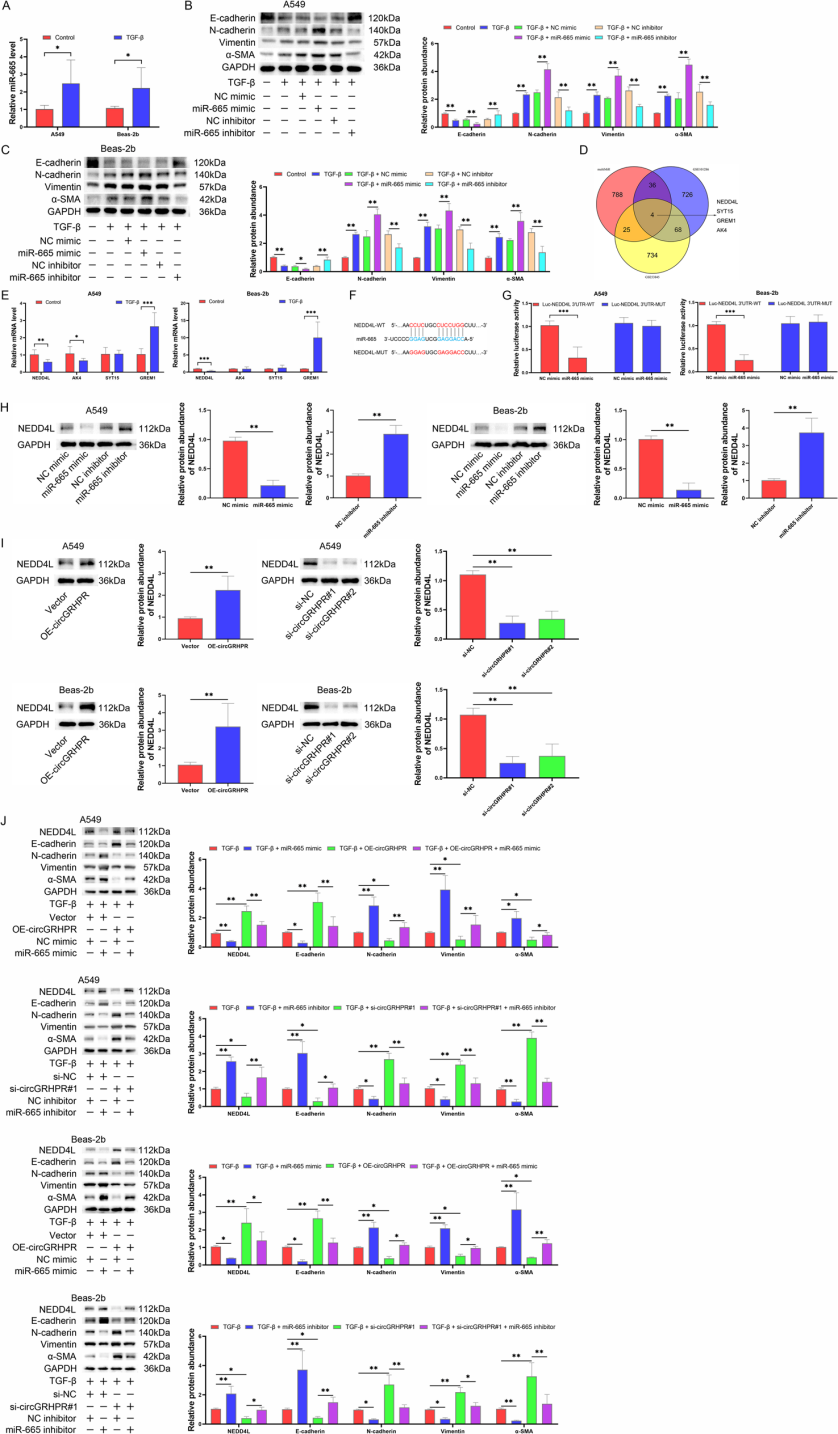
circGRHPR/miR-665 targets NEDD4L to regulate EMT progression of TGF-β1-induced LECs in vitro. **A** The expression of miR-665 in TGF-β1-induced LECs. **B** The effect of miR-665 on EMT progression in A549. **C** The effect of miR-665 on EMT progression in Beas-2b. **D** Prediction results of downstream target genes of miR-665. **E** The expressions of potential target genes in TGF-β1-induced LECs. **F** The sequences of binding site between NEDD4L and miR-665 in dual-luciferase reporter assays. **G** The results of dual-luciferase reporter assays of NEDD4L and miR-665 in LECs. **H** The effect of miR-665 on NEDD4L expression in LECs. **I** The effect of circGRHPR on NEDD4L expression in LECs. **J** The effect of circGRHPR and miR-665 on NEDD4L expression and EMT progression in TGF-β1-induced LECs. All data were reported as mean ± SD. Significant levels were indicated as *P < 0.05, **P < 0.01, ***P < 0.001

We then explored the function of miR-665. Firstly, we found that the abnormal EMT progression of TGF-β1-induced LECs was accompanied by upregulation of miR-665 (Fig. 4A). The results of western blot showed that transfection of miR-665 mimic promoted the abnormal EMT progression of TGF-β1-induced LECs in vitro, while transfection of miR-665 inhibitor significantly inhibited the progression of EMT (Fig. 4B, C).

We used the multiMiR package of R software to predict the target genes of miR-665 and further screened the results using microarray data GSE101286 and GSE53845. Four common genes (NEDD4L, SYT15, GREM1, AK4) were identified in all three datasets (Fig. 4D). We found that the downregulation of NEDD4L was associated with abnormal EMT progression of TGF-β1-induced LECs (Fig. 4E), which was negatively correlated with the transcription level of miR-665. It suggested that NEDD4L might be the downstream target gene of miR-665. The results of dual-luciferase reporter assay showed that miR-665 mimic significantly inhibited luciferase activity of NEDD4L-WT luciferase reporter plasmid, but not the NEDD4L-MUT reporter (Fig. 4F, G), and the NEDD4L protein expression was negatively regulated by miR-665 and positively regulated by circGRHPR (Fig. 4H, I). Furthermore, we found that the effect of circGRHPR on inhibiting the abnormal EMT progression of TGF-β1-induced LECs was reversed by miR-665 mimic, which was accompanied by the downregulation of NEDD4L. Conversely, we found that miR-665 inhibitor delayed EMT progression caused by circGRHPR downregulation, which was related to the upregulation of NEDD4L (Fig. 4J).

### Reversing the expression of NEDD4L counteracted the effect of circGRHPR on EMT progression in TGF-β1-induced LECs

**Fig. 5:**
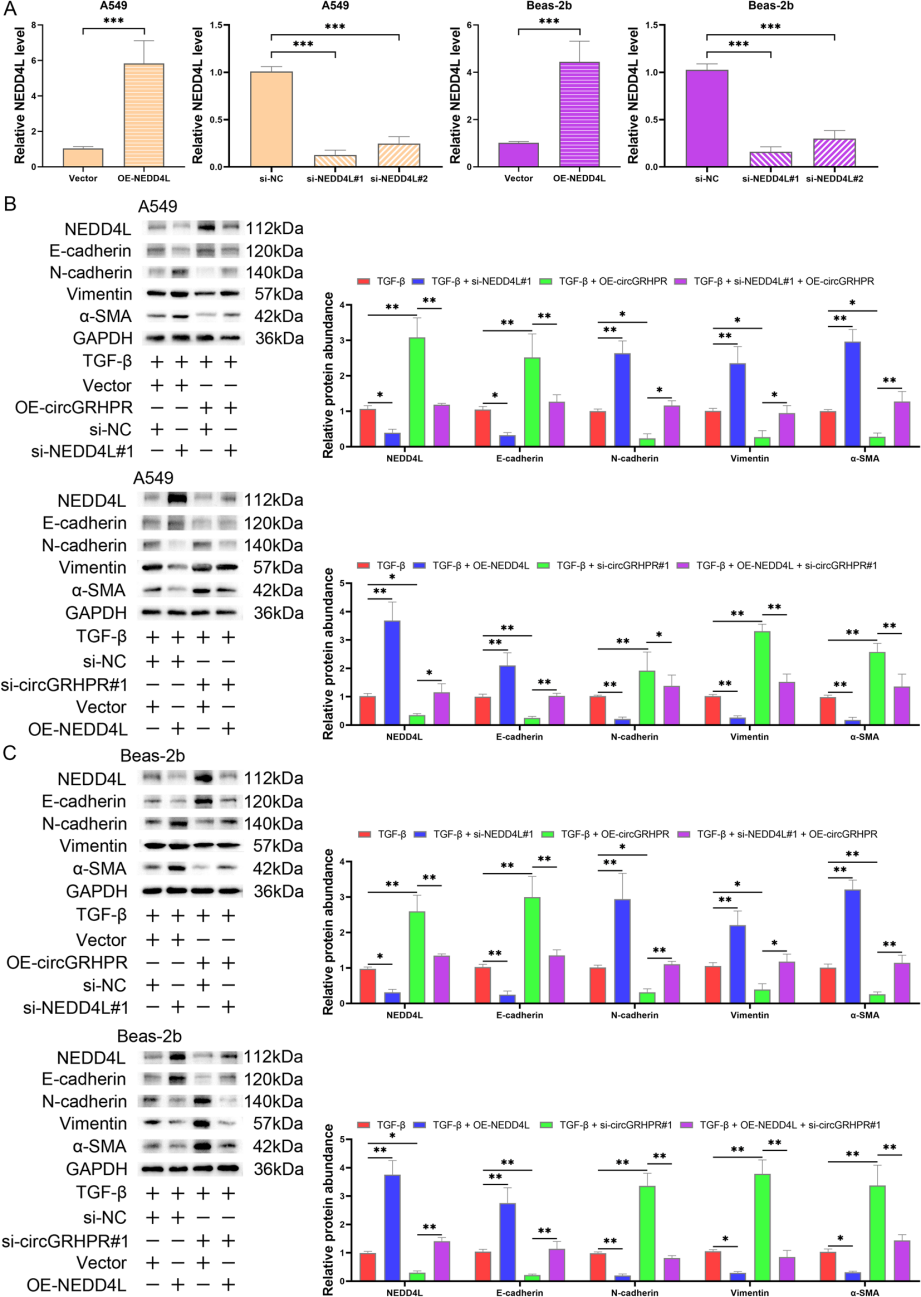
Effects of circGRHPR and NEDD4L on abnormal EMT progression of TGF-β1-induced LECs. **A** The transfection efficiency of NEDD4L overexpressed plasmid and siRNA in LECs. **B** The effect of circGRHPR and NEDD4L on abnormal EMT progression in A549s. **C** The effect of circGRHPR and NEDD4L on abnormal EMT progression in Beas-2b. The data was expressed as mean ± SD. *P < 0.05, **P < 0.01, ***P < 0.001

Recent studies have shown that the deletion of NEDD4L in alveolar epithelial cells leads to progressive IPF in mice (Duerr et al. 2020). We first evaluated the transfection efficiency of NEDD4L (Fig. 5A). In order to ascertain the impact of circGRHPR on EMT progression through the miR-665/NEDD4L axis, we assessed whether regulating the expression of NEDD4L can reverse the function of circGRHPR in LECs. As the results showed, inhibiting circGRHPR on EMT progression was reversed by the downregulation of NEDD4L in TGF-β1-induced LECs. When NEDD4L was upregulated, the promoting effect of downregulated circGRHPR on EMT progression was reversed (Fig. 5B, C).

### circGRHPR enhances the ubiquitination of TGFBR2 in LECs

**Fig. 6:**
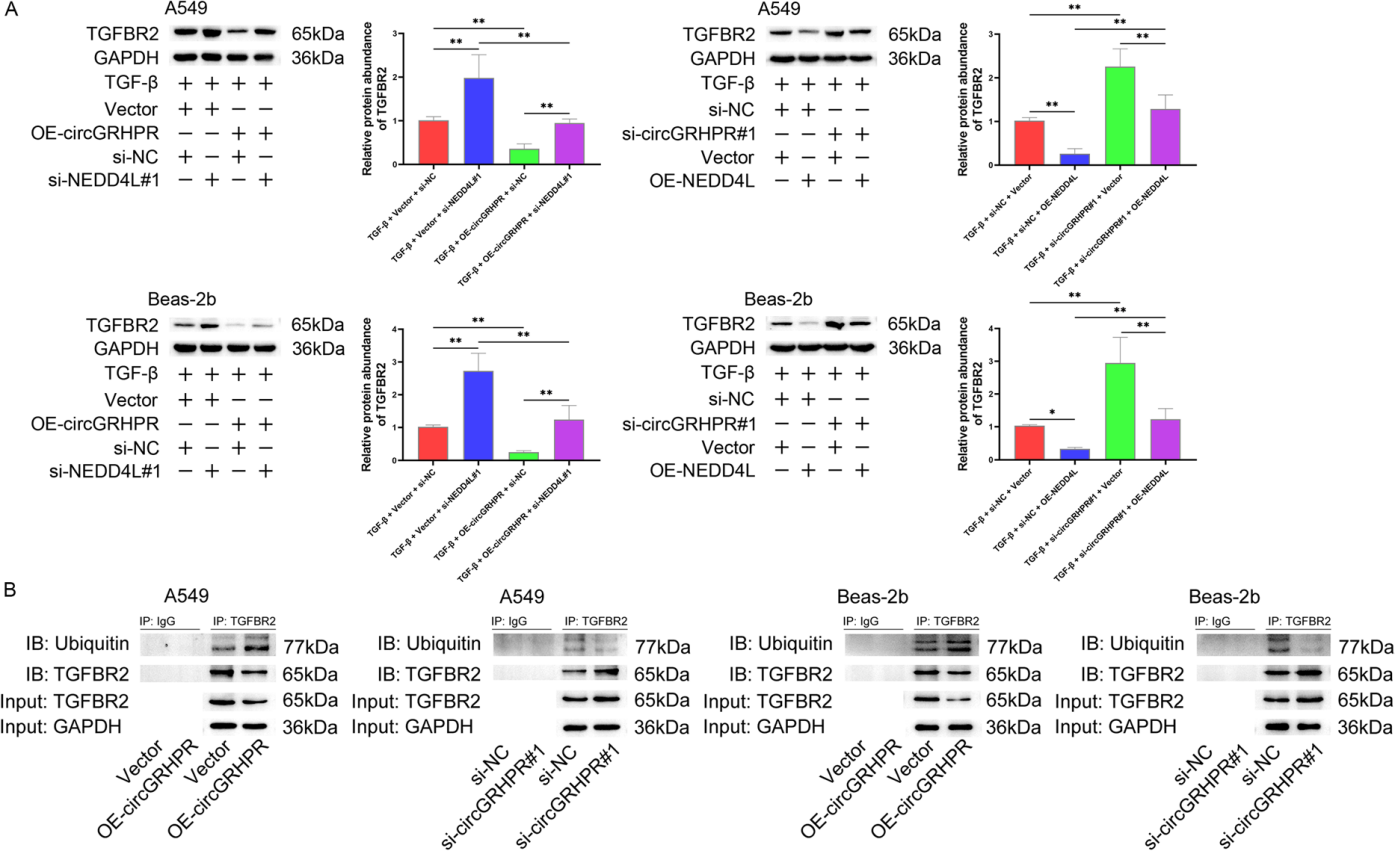
circGRHPR regulates the expression of TGFBR2 by mediating the degree of its ubiquitination in LECs. **A** The effect of circGRHPR on NEDD4L-mediated TGFBR2 expression. **B** Co-IP analysis of the effect of circGRHPR on the ubiquitination degree of TGFBR2. Data was presented as mean ± SD. *P < 0.05, **P < 0.01

NEDD4L alleviates experimental pulmonary fibrosis by targeting ubiquitination of TGFBR2 in lung fibroblasts (Li et al. 2023). We found that circGRHPR affected the regulation of NEDD4L on TGFBR2 expression in LECs (Fig. 6A). We further observed that this phenomenon was associated with the regulation of TGFBR2 ubiquitination, which is mediated by circGRHPR (Fig. 6B).

## Discussion

The pathogenesis of IPF is intricate and the precise mechanism remains unclear, which makes its treatment extremely difficult. Most previous studies have suggested that excessive proliferation and activation of myofibroblasts are the core mechanism of IPF (Hettiarachchi et al. 2020; Matera et al. 2020). However, recent studies have shown that LEC injury, including alveolar and bronchial epithelial cells, may be the key factor for the deterioration of IPF (Gu et al. 2022; Zhang et al. 2022; Veith et al. 2021). In more detail, alveolar epithelial cells are thought to contribute to IPF by producing myofibroblasts through abnormal EMT progression (Song et al. 2011; Kim et al. 2009). Meanwhile, bronchial epithelial cells also exacerbate airway remodeling of IPF through abnormal EMT progression (Wang et al. 2023). EMT is a phenomenon where epithelial cells undergo a transformation, transitioning from their original characteristics to adopt the traits of interstitial cells (Fontana et al. 2023; Zhang et al. 2021). The main manifestations of EMT are downregulation of E-cadherin representing epithelial components and upregulation of N-cadherin representing interstitial components. Some studies have proved that circRNAs are involved in the progress of EMT in mammary epithelial cells and gastric mucous epithelial cells (Kurosaki et al. 2021; Li et al. 2022). Up to now, there is no evidence to show whether circRNAs can participate in the mechanism of IPF by regulating the EMT progression of LECs. In both in vivo and in vitro models of IPF, overactivated TGF-β1 signal is the central regulator of abnormal EMT progression of LECs (Inui et al. 2021; Andugulapati et al. 2020). According to this theory, we found for the first time that the downregulation of circGRHPR was associated with TGF-β1-induced EMT progression of LECs *in vitr*o. In this context, A549 cells represent alveolar epithelial cells while Beas-2b cells represent bronchial epithelial cells (Kim et al. 2019; Schuliga et al. 2021; Li et al. 2020b). At the same time, after analyzing the peripheral blood samples, we also observed a notable downregulation of circGRHPR in individuals with IPF compared to those without any health conditions. Overall, circGRHPR has the potential to serve as a novel biomarker for aiding in the diagnosis of IPF and is associated with the abnormal EMT progression of LECs in its pathological course.

As a constituent of the non-coding RNA family, circRNAs are stably expressed in human tissues, including the lungs. In most cases, circRNAs are incapable of direct encoding and translation into proteins, necessitating their functional realization through diverse downstream mediators (Mirzaei et al. 2022). According to existing studies, circRNAs show different subcellular localizations in different kinds of diseases. Some circRNAs are localized in the nucleus and function through interactions with RNA-binding proteins (Wei et al. 2023). The majority of circRNAs are predominantly found in the cytoplasm and play a regulatory role by sponge miRNAs, thereby modulating the expression of downstream target genes (Papatsirou et al. 2021). In more detail, the presence of miRNA-targeting binding sites in circRNAs forms the foundation for its adsorption as a sponge. As the most common mediators of circRNAs, several studies have found the involvement of miRNAs in fibroblast-mediated progression of IPF (Rackow et al. 2022; Bahudhanapati et al. 2019). However, the precise role of miRNA in EMT progression of LECs remains elusive. In this study, we found that circGRHPR was mainly localized in the cytoplasm of LECs and inhibited TGF-β1-induced EMT progression by sponging miR-665. Meanwhile, the expression of miR-665 was found to be significantly upregulated in TGF-β1-induced LECs. Liu et al. also confirmed that the overexpression of miR-665 promoted the progression of liver fibrosis (Liu et al. 2022). In contrast, we noted that transfection with miR-665 inhibitors effectively impedes EMT progression of TGF-β1-induced LECs. This suggests that the upregulation of miR-665 may be related to IPF. However, the expression level, diagnostic value, and clinical significance of miR-665 in IPF patients need to be further explored.

Similar to circRNAs, miRNAs belong to the non-coding RNA family. In contrast to circRNAs, miRNAs specifically bind to the 3′-UTR region of downstream target mRNAs, thereby facilitating mRNA degradation or suppressing their protein translation (Fu et al. 2023). In this research, NEDD4L was identified as a downstream target gene regulated by miR-665. We observed a notable decrease in the expression of NEDD4L during the EMT progression of TGF-β1-induced LECs. In line with our findings, Ling et al. confirmed that silencing NEDD4L promoted EMT progression in TGF-β1-induced LECs and aggravated IPF (Ling et al. 2022). Our study further revealed that circGRHPR exhibited the ability to reverse the regulatory impact of miR-665 on NEDD4L expression. More importantly, we found in the rescue experiment that interfering with NEDD4L expression directly attenuates the protective effect of circGRHPR on EMT progression in TGF-β1-induced LECs. These results proved that the circGRHPR/miR-665/NEDD4L axis regulated the abnormal EMT progression of LECs.

We further explored the mechanism by which the circGRHPR/miR-665/NEDD4L axis regulates the progression of abnormal EMT in LECs. According to the results of previous studies, NEDD4L plays an anti-inflammatory and anti-fibrotic role in IPF by targeting ubiquitination and degradation of TGFBR2 (Li et al. 2023). The signaling pathway of TGF-β1 plays a pivotal function in the advancement of IPF (Boutanquoi et al. 2020). TGFBR2 serves as a crucial participant in the transmission of TGF-β1-mediated signal and together with TGFBR1 constitutes the TGF-β1 receptor complex located on the cell surface (Hinck 2012). TGFBR2 initially recognizes and binds to TGF-β1, thereby phosphorylating TGFBR1 and subsequently activating the SMAD-related pathway, which contributes to the progression of IPF (Jacko et al. 2016). We observed that circGRHPR affected the regulatory effect of NEDD4L on TGFBR2 expression, concomitant with alterations in TGFBR2 ubiquitination levels. These above findings indicated that the circGRHPR/miR-665/NEDD4L axis exerts inhibitory effects on the EMT progression of TGF-β1-induced LECs by facilitating the ubiquitination of TGFBR2 in vitro.

There are certain constraints associated with this study. The current challenge lies in acquiring lung tissue from IPF patients for clinical trials due to limitations in transplantation eligibility. Additionally, the absence of a homologous gene for circGRHPR in mice and rats restricts the development of animal experiments. Although we found that the downregulation of circGRHPR in peripheral blood of patients was associated with the diagnosis of IPF, however, we cannot currently demonstrate that circGRHPR is involved in the abnormal EMT progression of LECs in vivo. At present, we focused on establishing a connection between clinical phenomena with the phenotype of LECs in vitro. We found that the circGRHPR/miR-665/NEDD4L axis ameliorated the EMT abnormal progression of TGF-β1-induced LECs by promoting TGFBR2 ubiquitination in vitro. This mechanism casts a light on understanding the onset and progression of IPF.

## Conclusion

Together, we have showed for the first time that circGRHPR was downregulated in peripheral blood of patients with IPF. Meanwhile, circGRHPR inhibited the abnormal EMT progression of TGF-β1-induced LECs in vitro, which mimics the alteration of LECs in IPF patients. The function of circGRHPR mainly depends on the regulation of TGFBR2 ubiquitination by miR-665 and NEDD4L. In summary, targeting the circGRHPR/miR-665/NEDD4L axis may hold promise as a therapeutic strategy for treating IPF patients in the future clinical interventions.

## Data Availability

All data generated or analyzed during this study are included in this published article.
